# Assessment of Inter- and Intrasurgeon Variability in Preoperative Planning of Reverse Shoulder Arthroplasty: A Multicenter Evaluation

**DOI:** 10.1055/s-0044-1788783

**Published:** 2024-08-16

**Authors:** Geraldo da Rocha Motta Filho, Marcus Vinicius Galvão Amaral, Luis Gustavo Prata Nascimento, André Couto Godinho, Caio Santos Checchia, Mauricio de Paiva Raffaelli, Rafael Peçanha Pitta, Ana Carolina Leal

**Affiliations:** 1 Centro de Cirurgia do Ombro e Cotovelo, Instituto Nacional de Traumatologia e Ortopedia Jamil Haddad, Rio de Janeiro, RJ, Brasil; 2 Divisão de Ensino e Pesquisa, Instituto Nacional de Traumatologia e Ortopedia Jamil Haddad, Rio de Janeiro, RJ, Brasil; 3 Divisão de Ortopedia e Traumatologia, Instituto Nacional de Traumatologia e Ortopedia Jamil Haddad, Rio de Janeiro, RJ, Brasil; 4 Serviço de Cirurgia do Ombro e Cotovelo, Faculdade de Medicina do ABC, São Paulo, SP, Brazil; 5 Serviço de Cirurgia do Ombro do Hospital Ortopédico, Belo Horizonte, MG, Brasil; 6 Faculdade de Medicina, Universidade de São Paulo, São Paulo, SP, Brasil; 7 Instituto Naeon – Núcleo Avançado de Estudos em Ortopedia e Neurocirurgia, São Paulo, SP, Brasil; 8 Hospital da Força Aérea do Galeão – HFAG, Rio de Janeiro, RJ, Brasil

**Keywords:** arthroplasty, replacement, shoulder/methods, observer variation, shoulder joint/surgery, software

## Abstract

**Objective** To evaluate the intra and intersurgeon variability regarding the positioning and selection of implants in reverse shoulder arthroplasty.

**Methods** A cross-sectional study assessed computed tomography images of the shoulder joint of patients diagnosed with degenerative joint diseases. The study team included seven specialists in shoulder surgery, representing six different institutions. Surgeons were instructed to plan all cases twice, and then we evaluated inter- and intrasurgeon variability.

**Results** The interclass correlation for version and inclination showed low agreement concerning inclination (0.26), and moderate agreement for version (0.73) and graft selection (0.54). The intrasurgeon evaluation revealed a moderate correlation for version (0.55), inclination (0.58), and implant selection (0.46), while for lateralization the correlation was high (0.77).

**Conclusion** This comparative study of preoperative planning by different surgeons showed the lack of consensus on implant positioning parameters during reverse shoulder arthroplasty planning. However, most surgeons tend to plan for zero degrees of version and inclination.

## Introduction

Determining the glenoid version and inclination is critical for arthroplasty planning and execution since joint deformities require correction before component implantation. 1 Poor glenoid component positioning, with excessive retroversion, inclination, or both, predisposes to instability and loosening, impacting the range of motion. 2 3 4 5 6 7

Preoperative planning of shoulder arthroplasties can be performed through automated programs that identify morphological changes and allow the surgeon to correct existing deformities and select the ideal implants. 7 8 As a result, surgeons anticipate peculiarities of the surgical technique, potentially improving the precision in implant positioning and impacting outcomes.

Despite such technologies, glenoid deformity correction and positioning are subjective since the need to consolidate arthroplasty parameters for better outcomes remains. 8 9 10 11 There is minimal clinical evidence to establish an ideal version and inclination range or the clinical manifestations potentially resulting from a deviation from this range. 12 13 Thus, planning occurs based on the surgeon's concepts, preferences, and personal experiences, leading to inter- and intraobserver discrepancies when planning the same case. 8 14

The present study aimed to evaluate inter- and intrasurgeon variability in the following aspects of preoperative planning for reverse total shoulder arthroplasty (RTSA): version and inclination correction, selection of metal base characteristics, bone graft use or not, and the consequent lateralization and distalization of the glenoid component. The hypotheses are that multiple surgeons will plan the same case with intersurgeon variability and that planning on separate occasions will reveal intrasurgeon variability. 12 13

## Materials and Methods

After approval by the Institutional Research Ethics Committee (opinion no. 35243920.4.0000.5273), a cross-sectional study evaluated shoulder joint computed tomography (CT) images.

The study team consisted of 7 shoulder surgery specialists representing 6 different institutions, all of whom had more than 10 years of clinical experience and knowledge using the selected automated platform.

All CT scans occurred at the main author's home institution, with the patient in the supine position and using a 64-channel Brilliance equipment (Philips, Amsterdam, Netherlands), with 1-mm slices. The study included CT scans of patients from both genders, aged over 18, with primary or secondary degenerative disease of the shoulder regardless of the glenoid or humeral head deformity degree. We excluded patients with other diagnoses, previously subjected to shoulder surgeries, and whose imaging tests showed changes hindering the processing by the selected software.

We coded the imaging tests to preclude identification and provided no clinical information of the patients. We asked the surgeons to plan the cases without specific guidance, that is, each surgeon defined their strategy using their own criteria.

Planning was performed using the Blueprint software (Tornier SAS, Saint Martin, France), which performs the segmentation, reformatting, and three-dimensional (3D) reconstruction, in addition to automated glenoid version and inclination measurements.

The software allowed the selection of a metal base with 2 diameters, 25 and 29 mm, and the definition of its positioning. Furthermore, the surgeon assessed the potential need for glenoid reconstruction using bone grafts, which could be symmetrical, with a thickness of 7 mm or 10 mm, or asymmetrical, with 12.5° in angulation and 10 mm in thickness. Next, the surgeon selected the glenosphere, which comes in 2 different diameters, 36 and 42 mm, and which could be centric, with 2 mm of lower eccentricity, or with a 10° lower inclination.

A short metaphyseal fixation humeral rod was selected in relation to its diameter and positioning. Its polyethylene composition and thickness were always the same, and a medialized humeral tray was used in all cases.

After a minimum period of four weeks, we asked the surgeons to replan each case without access to the planning previously performed. A researcher not involved in the analyses sent the cases to the surgeons and monitored the time between the first and second planning. This monitoring allowed to keep the planning intervals homogeneous between evaluators.

The results were tabulated in specific electronic forms, namely, Google Forms, allowing the information from each plan to be attached and sent to another researcher for its blind evaluation.

## Statistical Analysis

All analyses were performed with the GraphPad Prism version 8.0 (GraphPad Software, LLC, Boston, MA, EUA) or MedCalc (MedCalc Software Ltd., Washington, DC, EUA) software. Interclass correlation coefficients were used to determine intersurgeon variability for continuous version, inclination, and lateralization data, considering each assessment round as an independent sample. The Kappa coefficient determined intersurgeon variability for categorical variables (base type and graft selection). Pearson correlation coefficients specified intrasurgeon variability for continuous variables, that is, version, inclination, and lateralization between the two planning rounds. We presented the data as mean ± standard deviation followed by minimum and maximum values.

## Results

**Fig. 1:**
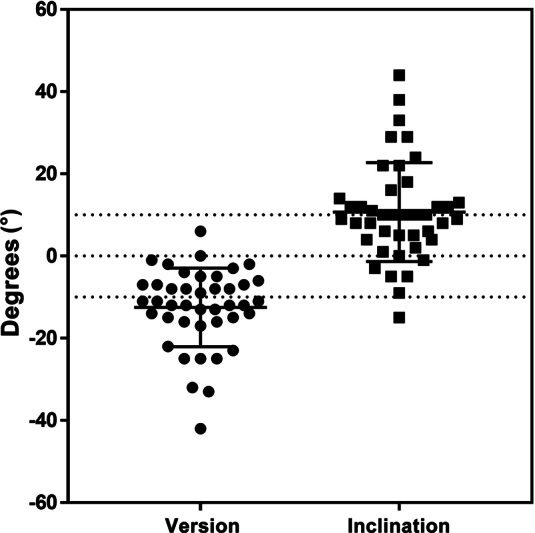
Version and inclination values in the cases analyzed.

We evaluated 42 cases, with 21 being rotator cuff arthropathy and 21 osteoarthritis. The mean preoperative version of the evaluated cases was −12.5° ± 9.6° (minimum: −42°; maximum: 6°) and the mean inclination was 10.7° ± 12° (minimum: −15°; maximum: 44°) ( Fig. 1 ).

**Fig. 2:**
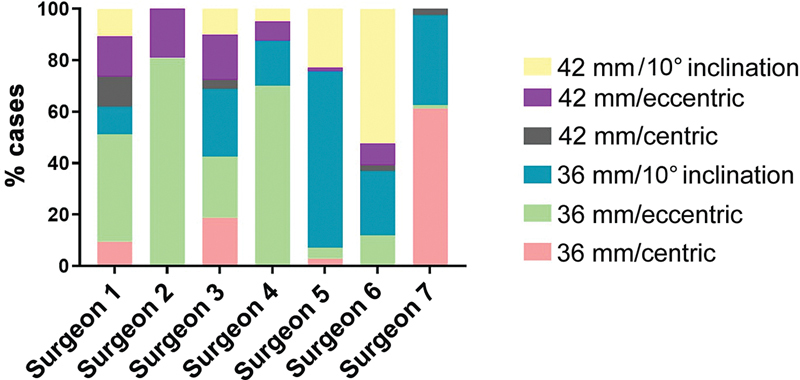
Histogram representing the percentage of use of each glenosphere type by surgeons.

Regarding planning, 76% of cases (61–98%) chose the 25-mm diameter metal base. The glenosphere selection occurred as follows: 36-mm eccentric device in 33% (1–80%), the 36-mm device with a lower inclination of 10° in 26% (0–68%), the 36-mm centric device in 13% (0–61%), the 42-mm device with a lower inclination of 10° in 15% (0–52%), the 42-mm eccentric device in 10% (0–19%), and the 42-mm device in 3% (0–12%) of the cases ( Fig. 2 ).

**Fig. 3:**
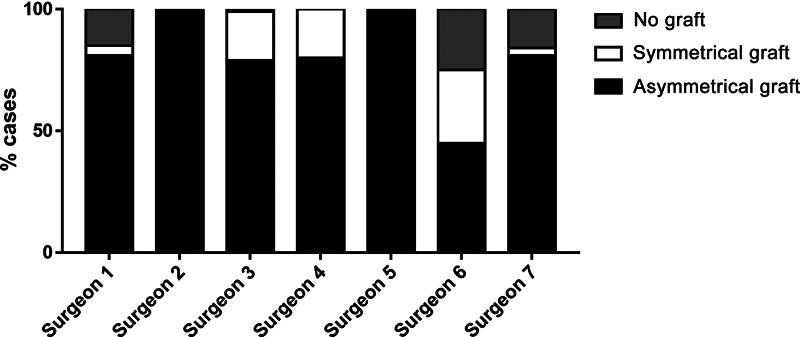
Histogram representing the percentage of grafts used by surgeons.

Eighty percent of the plannings used a 10-mm asymmetrical graft with 12.5° of inclination, while 11% employed a symmetrical graft, and only 9% did not plan for grafts ( Fig. 3 ).

**Fig. 4:**
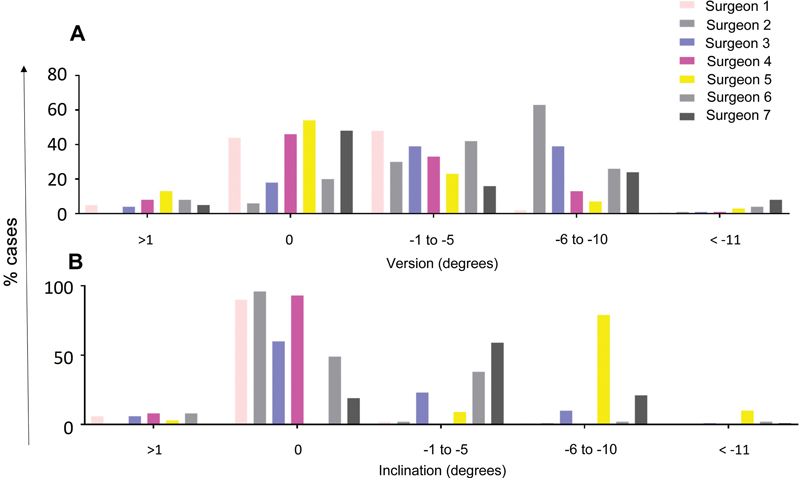
Distribution of the final planning of ( **A** ) version and ( **B** ) inclination among surgeons.

**Fig. 5:**
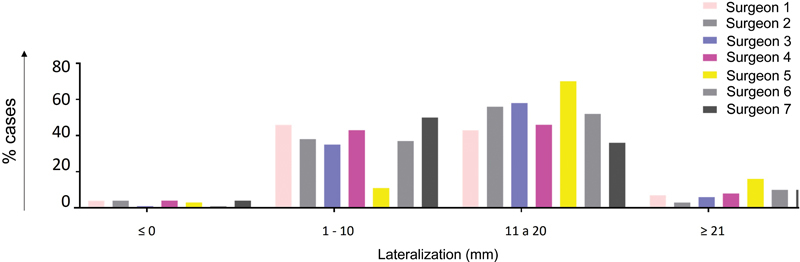
Schematic figure of the postoperative version of the evaluated cases planned by each surgeon in the two rounds.

As for version planning, 34% of the cases would have a final version of 0° (6–54%), 33% included a postoperative version ranging from −1° to −5° (16–48%), and for 25% (4–64%), it included a final version ranging from −6° to −10° ( Fig. 4A ). Only 5% of cases (0–13%) intended for positive version values, and an even smaller number of cases, 3% (1–8%), aimed for retroversion values higher than −10°. Fig. 5 shows the final version intervals planned by each surgeon in the two rounds for all cases analyzed.

Regarding inclination, 58% of cases intended a final angulation equal to 0° (0–96%); this angulation ranged from −1° to −5° in 19% (0–59%) of cases and from −6° and −10° in 16% of cases (1–79%). Only 4% of cases intended a final positive inclination > 1° (0–8%), while 2% aimed for values lower than −11° (0–10%). As for inclination, it is worth highlighting that one of the surgeons did not plan any of the cases for a final inclination of 0°. Excluding this surgeon, 89% (77–98%) of cases intended to achieve a final inclination from 0° to −5°. Two surgeons planned most cases (97% and 81%) for a final inclination lower than −1° ( Fig. 4B ).

**Fig. 6:**
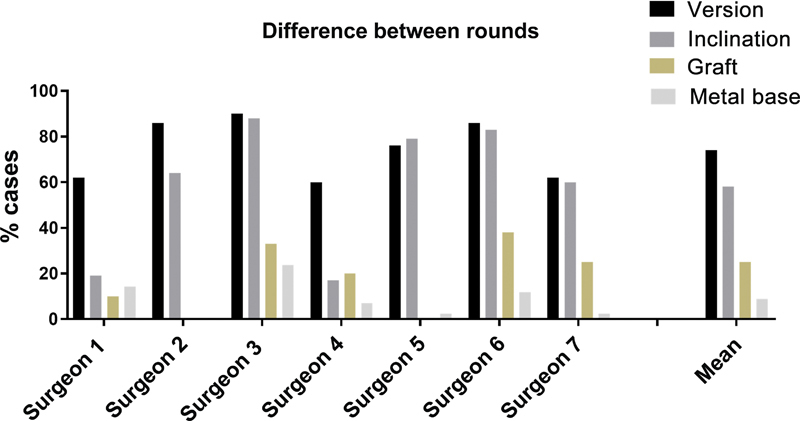
Distribution of the final lateralization planned per surgeon.

In most planned cases (52%), the intended lateralization ranged from 11 to 20 mm. In 37% (11–50%) of cases, lateralization ranged from 1 to 10 mm; in 8% (3–16%), it was higher than 21 mm, and it was equal to or lower than 0 mm in only 3% (1–4%) of the cases ( Fig. 6 ).

Table 1 presents the interclass correlation values for version and inclination. Interestingly, despite a moderate agreement for version (0.73), the agreement between different evaluators was low for inclination (0.26). The agreement between evaluators was also moderate for graft type selection (0.54).

**Table 1:** Interclass correlation coefficient

Variable	Coefficient	95% confidence interval
Version (ICC)	0.73	0.629–0.822
Inclination (ICC)	0.26	−0.04–0.501
Lateralization (ICC)	0.94	0.922–0.965
Graft*	0.54	0.45–0.62

**Abbreviation:** ICC, Interclass correlation coefficient.

**Note:** *Kappa coefficient.

**Fig. 7:**
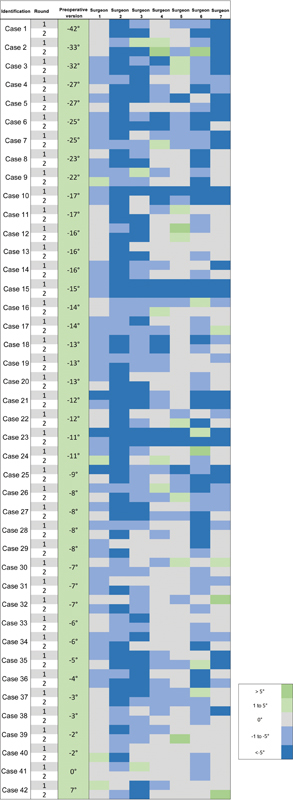
Percentage of cases with different version planning, metal base inclination, and graft use between the two rounds.

An analysis of the two planning rounds showed that surgeons planned different final versions in 74% (60–90%) and different inclinations in 58% of cases (17–88). The choice of the metal base diameter, 25 or 29 mm, also varied between rounds in 25% of cases (0–38%) ( Fig. 7 ).

The average difference between planning rounds was 0.98° for version and 1.8° for inclination. The difference ranged from 1° to 5° in 44% of cases (19–55%) and from 5° to 10° in 13% (5–24%), and it was higher than 10° in 16% (2–48%) of cases.

Table 2 shows the Pearson correlation coefficient for version, inclination, lateralization, and the agreement in graft selection. The correlation between rounds was moderate for version (0.55) and inclination (0.58), and high for lateralization (0.7).

**Table 2:** Assessment of the planning agreement of version, inclination, lateralization, and implant choice between the two rounds

Variable	Coefficient	95% confidence interval
Version	0.48	0.387–0.567
Inclination	0.59	0.512–0.664
Lateralization	0.77	0.717–0.82
Graft*	0.467	0.352–0.581

**Notes:** Intrasurgeon agreement (Pearson); *Kappa coefficient.

## Discussion

There is still no standardization regarding the anatomical parameters recommended for implant positioning in RTSAs. As such, surgeons must adopt individual criteria based on their experience and training when planning and performing the procedure. 14 This subjectivity can lead to significant variability between surgeons and different planning of the same case by a surgeon. Therefore, this multicenter study tried to evaluate intra- and intersurgeon variability in RTSA planning.

The metal base can impact RTSA outcomes since the size incompatibility between the glenoid and this component may alter the postoperative range of motion. 15 In the present study, surgeons selected the 25-mm metal base in 76% of cases. Since the glenoid size depends on the patient's ethnicity and gender, 16 the same factors may influence the choice of the metal base. However, we cannot say that gender influenced the choice of surgeons since the patients' clinical information was unavailable. Furthermore, a biomechanical study showed that 25-mm bases have less micromovement and a higher impact-free range of motion than 29-mm bases, 17 which may also have influenced the preference for this base size.

As for the glenosphere, it is noteworthy that most surgeons opted for an eccentric implant regardless of its size. This choice may have occurred because recent studies showed that eccentricity seems associated with better deltoid muscle efficiency despite the glenosphere size, resulting in a higher range of movement, especially for adduction. 18 19

Glenoid deformities require treatment for the correct positioning of the metal base and the complete introduction of the central pin into the bone mass, improving implant fixation and stability. Deformity correction may employ milling, bone grafts, or enlarged metal components. In our study, the system only allowed for the first two options. The results showed that surgeons selected graft in most cases (91%), preferably asymmetrical (80%). Cases including bone grafts had more severe bone deformities, with an average version of −13° and inclination of 11° compared to −8° and 6°, respectively, in cases planned with no bone graft. In the literature, other authors did not find a correlation between deformity severity and the influence on different plannings by different surgeons. 8 The bone graft was necessary to correct the deformities because, otherwise, it would imply excessive milling with a compromised bone stock. The bone graft corrects glenoid deformities to provide a greater lateralization of the entire system.

Regarding the final implant positioning in the glenoid, most surgeons aimed for 0° of the final version and inclination, consistent with the literature. 14 When analyzing the final version, 34% of cases were intended for 0° and 33%, for 1 to 5° of retroversion. Therefore, if we consider 5° as an acceptable residual deviation, 67% of cases were planned with a retroversion ranging from 0 to 5°. Planning included over 6° of retroversion in 25% of cases, over 10° in only 2%, and a final positive version in 6% of cases.

About the final inclination, 77% of the cases were planned for final versions ranging from 0 ^o^ to −5 ^o^ . Unlike the version, the tolerance for accepting a positive inclination, that is, superior, is much lower. This tolerance occurs because, in this orientation, arthroplasty may have complications, such as instability, component loosening, and consequent range of motion limitation. 11 20 In contrast, surgeons often desire a lower inclination, and our results revealed that 35% of cases presented an inferior inclination ranging from 1 to 10°, and in 2%, the inclination was above 10°. Therefore, in agreement with the literature, our results show a lack of consensus about the glenosphere inclination. 8 14 21 22

Regarding lateralization, for most cases (60%), the planning led to a final lateralization higher than 11 mm. This result is consistent with the findings of Bauer et al., who reported values between 13.1 and 35.8 mm. 23

Concerning intrasurgeon variability, we observed a difference between the first and second rounds in 74 and 58% of cases for version and inclination, respectively. Despite this, the average difference between the rounds was 0.98° for version and 1.8° for inclination, suggesting consistency in planning since these variations may have minimal or no clinical impact. The agreement on lateralization was high among surgeons, showing that different parameter combinations result in the same outcome.

This study has some limitations. Treatments occurred in a single reference center for high-complexity surgery. As such, the patients presented more severe deformities than those routinely found in clinical practice. Since the surgeons did not have access to the patient's clinical information, it is impossible to know the impact of these data on the surgeon's choices when planning RTSAs.

## Conclusion

This study on intra- and intersurgeon variability in RTSA planning highlights the lack of standardization guiding the ideal parameters for the procedure. Despite the variation, surgeons tend to plan the final version and inclination within a range of −5° to 5°, suggesting that different implant combinations and positioning patterns can lead to similar outcomes.
