# Supplementary material for: Assessment of Inter- and Intrasurgeon Variability in Preoperative Planning of Reverse Shoulder Arthroplasty: A Multicenter Evaluation
Source: Rev Bras Ortop (Sao Paulo). 2024 Aug 16;59(6):e950–7. doi: 10.1055/s-0044-1788783 (PMC11663061; doi:10.1055/s-0044-1788783)
Supplement: Related texts

# Research article: Avaliação da variabilidade inter e intracirurgião no planejamento pré-operatório da artroplastia reversa do ombro: Uma avaliação multicêntrica

**Keywords:** articulação do ombro/cirurgia, artroplastia do ombro/métodos, *software*, variações dependentes do observador

## Abstract

**Objetivo** Avaliar a variabilidade intra e intercirurgião em relação à seleção e posicionamento dos implantes na artroplastia total reversa do ombro (ATRO).

**Métodos** Foi realizado um estudo transversal de avaliação de imagens de tomografias computadorizadas da articulação do ombro de pacientes com diagnóstico de doenças articulares degenerativas. Participaram do estudo sete especialistas em cirurgia do ombro, representando seis diferentes instituições. Os cirurgiões foram instruídos a planejar todos os casos duas vezes e a variabilidade inter e intracirurgião foi avaliada.

**Resultados** A correlação interclasse para versão e inclinação apresentou uma concordância baixa em relação à inclinação (0,26), e moderada em relação à versão (0,73) e à seleção do enxerto (0,54). Na avaliação intracirurgião houve uma correlação moderada para versão (0,55), inclinação (0,58) e seleção do implante (0,46), enquanto para a lateralização, a correlação foi alta (0,77).

**Conclusão** Este estudo comparativo do planejamento pré-operatório da ATRO entre diferentes cirurgiões evidenciou que não há, ainda, um consenso em relação aos parâmetros de posicionamento dos implantes durante o planejamento da cirurgia. No entanto, a maioria dos cirurgiões tendem a planejar para uma versão e inclinação de zero grau.

## Introdução

A determinação da versão e da inclinação da glenoide é fundamental para o planejamento e execução da artroplastia, uma vez que para a implantação dos componentes é necessário corrigir as deformidades articulares. 1 O mau posicionamento do componente da glenoide, com excessiva retroversão e/ ou inclinação, predispõe a instabilidade e afrouxamento, além de impactar a amplitude de movimentos. 2 3 4 5 6 7

O planejamento pré-operatório das artroplastias de ombro pode ser realizado através de programas automatizados que identificam as alterações morfológicas e permitem que o cirurgião realize a correção das deformidades existentes e a seleção dos implantes a serem utilizados. 7 8 Dessa forma, os cirurgiões antecipam as peculiaridades da técnica cirúrgica, possivelmente aprimorando a precisão no posicionamento dos implantes e impactando os resultados

Apesar de tais tecnologias, a correção das deformidades e o posicionamento da glenoide ainda são realizados de forma subjetiva, uma vez que os parâmetros para realização das artroplastias associados aos melhores resultados ainda não estão consolidados. 8 9 10 11 Existem evidências clínicas mínimas para estabelecer uma faixa ideal da versão e inclinação, ou ainda quais seriam as manifestações clínicas que poderiam ocorrer quando existir desvio dessa faixa. 12 13 Dessa forma, os planejamentos são realizados a partir de conceitos, preferências e experiências pessoais do cirurgião, o que acarreta discrepâncias interobservador, e até mesmo intraobservador, em relação ao planejamento de um mesmo caso. 8 14

Este estudo tem como objetivo avaliar a variabilidade inter e intracirurgião nos seguintes aspectos do planejamento pré-operatório da artroplastia total reversa do ombro (ATRO): correção da versão e inclinação, seleção das características da base metálica, utilização ou não de enxerto ósseo e consequente lateralização e distalização do componente glenoidal. As hipóteses são as de que vários cirurgiões planejarão o mesmo caso com variabilidade intercirurgião e que o planejamento em ocasiões separadas, demonstrará variabilidade intracirurgião. 12 13

## Materiais e Métodos

Após aprovação do Comitê de Ética em Pesquisa Institucional (parecer n° 35243920.4.0000.5273), foi realizado um estudo transversal de avaliação de imagens de tomografias computadorizadas (TCs) da articulação do ombro.

Participaram do estudo 7 especialistas em cirurgia do ombro representando 6 diferentes instituições, que apresentavam experiência clínica superior a 10 anos, assim como experiência na utilização da plataforma automatizada selecionada.

Todas as TCs foram obtidas na instituição de origem do autor principal e realizadas com o paciente na posição supina em um tomógrafo Brilliance (Philips, Amsterdã, Holanda) de 64 canais, com cortes de 1 mm. Foram incluídas TCs de pacientes que apresentaram diagnóstico de doença degenerativa primária ou secundária do ombro, de ambos os sexos, com mais de 18 anos de idade, independentemente do grau de deformidade da glenoide ou da cabeça umeral. Foram excluídos os pacientes com outros diagnósticos, submetidos a cirurgias prévias do ombro e aqueles cujos exames de imagem apresentassem alterações que inviabilizassem o processamento pelo programa automatizado selecionado.

Os exames de imagem foram codificados de forma que não existisse nenhuma possibilidade de identificação, e nenhuma informação clínica do paciente foi fornecida. Os cirurgiões foram instruídos a planejar os casos sem nenhuma orientação específica, ou seja, cada cirurgião definiu sua estratégia utilizando critérios próprios.

Os planejamentos foram realizados no programa automatizado Blueprint (Tornier SAS, Saint Martin, França), que realiza o processo de segmentação e reformatação fornecendo a reconstrução em 3D, além de medições automatizadas da versão e inclinação da glenoide.

O programa permitiu a seleção da base metálica de 2 diâmetros, 25 e 29 mm, e a definição do seu posicionamento. Além disso, o cirurgião avaliou a eventual necessidade de reconstrução da glenoide utilizando ou não um enxerto ósseo, o qual poderia ser simétrico de 7 mm ou 10 mm, ou assimétrico com 12,5° de angulação e 10 mm de espessura. Em seguida, foi selecionada a glenosfera, existente em 2 diâmetros diferentes, 36 e 42 mm, e podendo ser cêntrica, com 2 mm de excentricidade inferior, ou ainda com 10° de inclinação inferior.

Foi selecionada a haste umeral curta de fixação metafisária em relação ao seu diâmetro e posicionamento, sendo a versão e a espessura do polietileno iguais em todos as situações e utilizada uma bandeja umeral medializada em todos os casos.

Após o mínimo de quatro semanas, os cirurgiões foram instruídos a replanejar cada um dos casos sem acesso ao realizado anteriormente. O envio dos casos para os cirurgiões assim como o acompanhamento dos tempos entre o primeiro e o segundo planejamento foi realizado por um pesquisador não envolvido nas análises. Dessa forma, foi possível manter o intervalo entre os planejamentos homogêneo entre os avaliadores.

Os resultados foram tabulados em formulário eletrônico específico (Google Forms), o que permitiu que as informações de cada planejamento fossem anexadas e enviadas a um outro pesquisador do estudo para serem avaliadas de forma cega.

## Análise Estatística

Todas as análises foram realizadas no software GraphPad Prism, versão 8.0 (GraphPad Software, LLC, Boston, MA, EUA), ou no software MedCalc (MedCalc Software Ltd., Washington, DC, EUA). Coeficientes de correlação interclasse foram usados para determinar a variabilidade intercirurgião para dados contínuos de versão, inclinação e lateralização, sendo que cada rodada de avaliação foi considerada uma amostra independente. O coeficiente Kappa foi utilizado para determinar a variabilidade intercirurgião para as variáveis categóricas (tipo de base e seleção do enxerto). Os coeficientes de correlação de Pearson foram usados para determinar a variabilidade intracirurgião para variáveis contínuas de versão, inclinação e lateralização entre as duas rodadas de planejamento. Os dados foram apresentados como média ± desvio padrão, seguido de mínimo e máximo.

## Resultados

Foram avaliados 42 casos, sendo 21 portadores de artropatia do manguito rotador e 21 de osteoartrite. A média da versão pré-operatória dos casos avaliados foi de −12,5° ± 9,6° (mínimo: −42°; máximo: 6°) e a média da inclinação foi 10,7° ± 12° (mínimo: −15°; máximo: 44°) ( Fig. 1 ).

**Fig. 1:** Valores de versão e inclinação mensurados nos casos analisados.

Em relação aos planejamentos, a base metálica de 25 mm de diâmetro foi escolhida em 76% dos casos (61-98%). Quanto à escolha da glenosfera, a de 36 mm excêntrica foi selecionada em 33% (1-80%) dos casos, a de 36 mm com inclinação inferior de 10° em 26% (0–68%), a de 36 mm cêntrica foi selecionada em 13% (0–61%), a de 42 mm com inclinação inferior de 10° em 15% (0–52%), a de 42 mm excêntrica em 10% (0–19%) e 42 mm cêntrica em 3% dos casos (0–12%) ( Fig. 2 ).

**Fig. 2:** Histograma representando o percentual de uso de cada tipo de glenosfera por cirurgião.

Em 80% dos planejamentos foi utilizado um enxerto assimétrico de 10 mm e 12,5° de inclinação, em 11% um enxerto simétrico e, em apenas 9% dos casos, não foi utilizado enxerto ( Fig. 3 ).

**Fig. 3:** Histograma representando o percentual de enxerto usado por cirurgião.

Em relação ao planejamento da versão, identificou-se que 34% dos casos foram planejados para uma versão final igual a 0° (6–54%), 33%, para versão pós-operatória variando entre −1° e −5° (16–48%), e 25% (4–64%), para versão final variando entre −6° e −10°. ( Fig. 4A ) Apenas 5% dos casos (0–13%) foram planejados para obter valores de versão positivos, e um número ainda menor de casos, 3% (1–8%), foram planejados para valores de retroversão superior a −10°. A Fig. 5 discrimina a versão final, categorizada em intervalos, planejada por cada cirurgião nos 2 rounds de planejamento para todos os casos analisados

**Fig. 4:** Distribuição do planejamento final da ( **A** ) versão e ( **B** ) inclinação entre os cirurgiões.

**Fig. 5:** Figura esquemática da versão pós-operatória dos casos avaliados, planejada por cada cirurgião nos dois rounds de planejamento.

Em relação à inclinação, 58% dos casos foram planejados para uma angulação final igual a 0° (0–96%), em 19% (0–59%) dos casos variando entre −1° e −5°, e em 16% (1–79%) dos casos variou entre −6° e −10°. Apenas 4% dos casos foram planejados de forma que inclinação final tivesse um valor positivo > 1° (0–8%) e, em apenas 2% dos casos, ela foi planejada para valores menores que −11° (0–10%). Em relação ao planejamento da inclinação, é importante ressaltar que um dos cirurgiões não planejou nenhum dos casos para uma inclinação final de 0°. Excluindo este cirurgião, 89% (77–98%) dos casos foram planejados para obter uma inclinação final variando entre 0° e −5°. Dois cirurgiões planejaram a maioria dos casos, 97% e 81%, para inclinação final menor que −1° ( Fig. 4B ).

Quanto à lateralização, foi observado que, na maioria dos casos planejados (52%) o resultado variou entre 11 e 20 mm. Em 37% (11–50%), a lateralização variou entre 1 e 10 mm, em 8% (3–16%), foi maior que 21 mm e, em apenas 3% (1–4%), foi igual ou menor que 0 mm ( Fig. 6 ).

**Fig. 6:** Distribuição do valor final de lateralização obtido para os planejamentos por cirurgião.

A Tabela 1 apresenta os valores de correlação interclasse obtidos para versão e inclinação. De forma interessante, ainda que tenha sido alcançada uma concordância moderada para a versão (0,73), em relação à inclinação, a concordância entre os diferentes avaliadores foi baixa (0,26). Também foi encontrada concordância moderada para a seleção do tipo do enxerto entre os avaliadores (0,54).

**Tabela 1:** Coeficiente de correlação interclasse

Variável	Coeficiente	IC 95%
Versão (CCI)	0,73	0,629–0,822
Inclinação (CCI)	0,26	−0,04–0,501
Lateralização (CCI)	0,94	0,922–0,965
Enxerto*	0,54	0,45–0,62

**Abreviaturas:** CCI, coeficiente de correlação interclasse; IC, intervalo de confiança.

**Nota:** *Índice Kappa.

Quando às duas rodadas de planejamento avaliadas, os cirurgiões planejaram versões finais diferentes entre si em 74% (60–90%) dos casos, e, em relação à inclinação, em 58% (17–88%). A escolha do diâmetro da base metálica, 25 ou 29 mm, também variou entre as rodadas em 25% (0–8%) ( Fig. 7 ).

**Fig. 7:** Percentual de casos em que o planejamento da versão, inclinação base metálica e uso de enxerto foi diferente entre os dois rounds.

A diferença média entre as rodadas de planejamento foi de 0,98° para versão e 1,8° para a inclinação. Em 44% (19–55%) dos casos a diferença variou entre 1 e 5°, em 13% (5–24%) entre 5 e 10° e em 16% (2–48%) foi maior que 10°.

A Tabela 2 mostra o coeficiente de correlação de Pearson para a versão, inclinação e lateralização, além da concordância na seleção do enxerto. Pode ser observada uma correlação moderada entre as rodadas, tanto para versão (0,55) quanto para inclinação (0,58), e uma alta correlação para a lateralização (0,7).

**Tabela 2:** Avaliação da concordância do planejamento de versão, inclinação, lateralização e escolha do implante entre os dois rounds de planejamento

Variável	Coeficiente	IC 95%
Versão	0,48	0,387–0,567
Inclinação	0,59	0,512–0,664
Lateralização	0,77	0,717–0,82
Enxerto*	0,467	0,352–0,581

**Abreviatura:** IC, intervalo de confiança.

**Notas:** Concordância intracirurgião (Pearson); *índice Kappa.

## Discussão

Ainda não existe uma padronização acerca dos parâmetros anatômicos recomendados para posicionamento dos implantes na ATRO, o que faz com que os cirurgiões adotem critérios individuais, com base em sua experiência pessoal e de formação para planejamento e realização do procedimento. 14 Esta subjetividade pode resultar em importante variabilidade entre diferentes cirurgiões assim como entre diferentes planejamentos de um mesmo caso por parte de um cirurgião. Dessa forma, este estudo buscou avaliar, através de um estudo multicêntrico, a variabilidade intra e intercirurgião no planejamento da ATRO.

A base metálica é um dos fatores que pode impactar nos resultados da ATRO, uma vez que a incompatibilidade de tamanho entre a glenoide e este componente pode impactar na amplitude de movimento pós-operatória. 15 No presente estudo, a base metálica de 25 mm foi selecionada em 76% dos casos. Uma vez que o tamanho da glenoide é influenciado pela etnia e pelo sexo do paciente, 16 a escolha da base metálica pode ser influenciada por estes mesmos fatores. Entretanto, não podemos afirmar que o sexo tenha influenciado a escolha dos cirurgiões, uma vez que as informações clínicas dos pacientes não foram disponibilizadas. Além disso, um estudo biomecânico evidenciou que as bases de 25 mm apresentam menor micromovimento e maior ADM livre de impacto que as de 29 mm, 17 o que também pode ter influenciado na preferência por este tamanho de base.

Quanto a glenosfera, chama a atenção o fato de que a maioria dos cirurgiões optaram por um implante excêntrico, independentemente do tamanho. O motivo de tal escolha pode ter sido o fato de estudos recentes terem evidenciado que excentricidade parece estar associada a melhor eficiência do músculo deltoide a despeito do tamanho da glenosfera, resultando em maior amplitude de movimento, principalmente adução. 18 19

As deformidades da glenoide precisam ser corrigidas para o posicionamento correto da base metálica, tanto em relação ao seu posicionamento, quanto ao objetivo de introduzirmos o pino central completamente dentro da massa óssea, melhorando a fixação e estabilidade do implante. A correção das deformidades pode ser obtida através de fresagem, utilizando-se enxerto ósseo ou com componentes metálicos com aumentos. No presente estudo, devido ao sistema utilizado, apenas as duas primeiras opções eram possíveis. Os resultados mostraram que os cirurgiões optaram pelo uso de enxerto na maioria dos casos (91%), sendo este preferencialmente assimétrico (80%). Os casos em que foram utilizados o enxerto ósseo apresentaram deformidades ósseas maiores, com versão média de −13 ^o^ e inclinação de 11 ^o^ em comparação com −8° e 6 ^o^ , respectivamente, nos pacientes cujo planejamento foi feito sem enxerto ósseo. Ao confrontarmos com a literatura, outros autores não encontraram correlação entre a gravidade da deformidade e a influência na diferença do planejamento entre diferentes cirurgiões. 8 O enxerto ósseo foi necessário para a correção das deformidades porque, de outra forma, implicaria em fresagem excessiva com comprometimento do estoque ósseo. O enxerto ósseo propicia, além da correção das deformidades da glenoide, uma lateralização maior de todo o sistema.

Em relação ao posicionamento final do implante na glenoide, a maioria dos cirurgiões objetivaram tanto a versão quanto a inclinação final em zero grau, assim como outros estudos na literatura. 14 Ao analisarmos a versão final, 34% dos casos foram planejados em 0 ^o^ , e outros 33%, entre 1° e 5° de retroversão. Portanto, se considerarmos 5° como desvio residual aceitável, 67% dos casos foram planejados entre 0 ^o^ e 5° de retroversão. Os casos com mais de seis graus de retroversão foram 25%, aqueles com mais de 10° apenas 2% e aqueles com versão final positiva 6%.

Em relação a inclinação final, 77% dos casos foram planejados para que a versão final estivesse entre 0 ^o^ e −5 ^o^ . Diferentemente da versão, a aceitação de uma inclinação positiva, ou seja, no sentido superior, é muito menos tolerada. Isto ocorre, pois nesta orientação a artroplastia apresenta-se associada a complicações, como instabilidade, soltura dos componentes e consequente limitação da amplitude de movimento. 11 20 Ao contrário, a inclinação inferior muitas vezes é desejada, e a análise dos resultados revelou que 35% ficaram de 1 a 10° inclinados inferiormente e 2% acima de 10°. Portanto, os resultados do presente estudo comprovam, assim como a literatura, que não há um consenso sobre a inclinação da glenosfera. 8 14 21 22

Quanto a lateralização, na maioria dos casos (60%) o planejamento resultou em lateralização final maior que 11mm. Este resultado é similar ao encontrado por Bauer e colaboradores que reportaram valores entre 13,1 e 35,8mm. 23

Quando a variabilidade intracirurgião foi avaliada, foi observada diferença entre o primeiro e segundo planejamento de 74% e 58% dos casos para versão e inclinação, respectivamente. Apesar disso a diferença média entre os rounds de planejamento foi de 0,98° para versão e 1,8° para inclinação, sugerindo uma consistência no planejamento, uma vez que estas pequenas diferenças, podem ter mínimo ou nenhum impacto clínico. Quanto a lateralização, houve elevada concordância entre os cirurgiões, evidenciando que diferentes combinações de parâmetros podem resultar em um mesmo desfecho.

Este estudo apresenta algumas limitações. Foram utilizados casos de pacientes atendidos em um único centro referência em cirurgia de alta complexidade, dessa forma, os casos apresentavam deformidades mais graves que as rotineiramente encontradas na prática clínica. Os cirurgiões não tiveram acesso a informações clínicas dos pacientes, dessa forma, não é possível saber o impacto dessas informações sobre as escolhas do cirurgião no planejamento da ATRO.

## Conclusão

Este estudo evidencia variabilidade intra e intercirurgião no planejamento da ATRO, evidenciando a ausência de uma padronização que oriente quanto aos parâmetros ideais para a realização do procedimento. Apesar da variação, os cirurgiões tendem a planejar a versão e inclinação final dentro de um intervalo entre −5° e 5°, sugerindo que diferentes combinações de implantes e padrões de posicionamento podem levar a desfechos similares.
